# Vision restoration in glaucoma: improved subjective visual function after personalised digital lifestyle coaching

**DOI:** 10.1007/s13167-026-00446-7

**Published:** 2026-03-14

**Authors:** Joana L. El Matine, Anke Lux, Luisa Fricke, Mahshid Tahamtan, Rami S. El Matine, Bernhard A. Sabel

**Affiliations:** 1 Institute of Medical Psychology, Medical Faculty, Otto-von-Guericke University of Magdeburg, Leipziger Straße 44, Magdeburg, 39120 Germany; 2 Institute of Biometry, Medical Faculty, Otto-von-Guericke University of Magdeburg, Leipziger Straße 44, Magdeburg, 39120 Germany; 3 Savir-Center GbR, Ulrichplatz 2, Magdeburg, 39104 Germany; 4 Institute for Diagnostic and Interventional Radiology, Medical Faculty, University of Bielefeld, Röntgenstraße 18, Detmold, 32756 Germany

**Keywords:** Patient-centred glaucoma care, Stress, Behavioural patterns, Health risks, Psychosocial factors, Digital health, Lifestyle intervention, Subjective visual function, Patient empowerment, Predictive preventive personalised medicine (PPPM / 3PM)

## Abstract

**Background:**

Glaucoma-related vision loss is associated with stress, anxiety, and reduced quality of life. Because availability of psychological support and evidence-based life style information for patients are limited, we developed “VisionWise” (ViWi), a personalised digital lifestyle education course for glaucoma patients. We hypothesized that ViWi participation could improve subjective well-being, increase knowledge about the disease, increase positive lifestyle habits and possibly improve subjective vision.

**Methods:**

In this exploratory AB/BA study (*N* = 27), patients of the AB group participated in a nine-week ViWi course (A) followed by a waiting period (B) while the BA group waited before taking the course. All were asked to complete questionnaires on stress (PSQ-20), depression (PHQ-8), anxiety (GAD-7), subjective vision (NEI-VFQ-25), personality (NEO-FFI), and glaucoma-related knowledge at three time points. Intervention effects were analysed as pre-post comparison: AB:T1–T2 and BA:T1–T3.

**Results:**

Subjective general vision improved significantly in group AB (*p* < .001) but also - unexpectedly - in group BA (*p* < .001) already during the waiting period, suggesting a placebo or expectancy effect. Changes were observed regarding stress-related measures, depressive symptoms, and anxiety. Some personality dispositions (higher agreeableness) were associated with greater reductions in anxiety and depressive symptoms. Usability ratings of ViWi were high, and no adverse events were observed.

**Conclusion:**

ViWi is a feasible and personalised digital lifestyle coaching tool to partially restore subjective vision in glaucoma patients. It can be used at home and empowers patients to cope with vision loss through behavioural eye exercises, management of stress-related risk factors, and novel strategies to adapt to low vision. Although the observed vision improvements are in part explainable by a placebo effect, this finding indirectly supports the relevance of positive attitudes in helping patients better control anxiety and stress, which are known co-factors in glaucoma progression. ViWi also supports patients in becoming better informed and more proactive in their glaucoma management. Our pilot study may guide future study designs to verify and optimise training effects through individualization, user engagement, and online accessibility as low-cost means “what else” they can do to complement standard glaucoma care. Within the framework of predictive, preventive and personalized medicine (PPPM), the present findings highlight the potential of digital lifestyle interventions to support patients´ awareness of psychosocial and stress-related risk factors, while exploratory analyses provide predictive insights into risk constellations associated with intervention outcomes. By addressing stress-related and behavioural factors, ViWi may contribute to a paradigm shift from reactive disease management towards a more personalised, positive, and patient-centred glaucoma care.

## Introduction

Glaucoma is an eye disease that affects not only the retina and optic nerve because of elevated intraocular pressure but also the brain [1], possibly because of vascular dysregulation [2]. However, despite glaucoma care using somatic interventions (pharmaceutical, surgical) vision loss typically still progresses, suggesting that other factors may also contribute. We recently proposed that there is a psychological/behavioural dimension to glaucoma progression, namely that mental stress also plays a critical role in the progression of vision loss, especially in persons with compulsive, perfectionistic personality traits which are a source of continuous stress [3–5]. This is supported by the observation that regular practice of relaxation exercises such as eye yoga or meditation can reduce stress hormone levels, normalize intraocular pressure and vascular dysregulation with subsequent visual field improvements [6–8]. Hence, psychological interventions could be a useful adjunct to standard glaucoma care. From the perspective of predictive, preventive and personalised medicine (PPPM), digital lifestyle interventions such as ViWi offer a promising approach to address psychosocial as well as behavioural risk factors relevant to glaucoma care, offering prevention strategies and support to glaucoma patients beyond the somatic treatment.

But psychological interventions are not part of today’s standard glaucoma care. Neither patients nor ophthalmologists are aware that healthy lifestyle and complementary treatments can complement glaucoma care because as of now little evidence-based information is available. Because methods are needed to provide easily accessible and cost-effective help [9] to improve patient care regarding lifestyle and mental health issues, an option in todays´ digital world is the potential use of a e-health learning platform.

We therefore developed and evaluated such an educational digital ehealth-platform, VisionWise (ViWi). Our goal was to educate patients about everyday behavioural and lifestyle factors that have the potential to slow down disease progression and possibly improve vision, e.g. by use of nutritional supplements or stress reduction techniques [6, 7, 10–13]. Here we describe an exploratory, prospective study to test if ViWi, an educational course, can improve vision-related knowledge, subjective visual performance, quality of life, psychological well-being, lifestyle, and subjective visual functions.

## Methods

This section describes the development of ViWi, its technical implementation, and the methodological framework of the study.

### Development of VisionWise

#### Content development

ViWi course modules were developed based on lifestyle factors known to be relevant for low vision patients. These modules and structured patient interviews focused on the following topics:

1. Lifestyle tips on stress management, exercise, sleep, behavioural advice, and eye exercises (eye yoga).
2. Nutritional advice, antioxidants and eye protection.
3. Coping with emotions and recognizing warning signs of deteriorating mental health at an early stage.
4. Understanding and coping with illness, dealing with lack of acceptance and grief, and the role of social support by their relatives.
5. Learning positive thinking, e.g. through affirmations.
6. Introduction to relevant internet aids, apps, or other media aimed at the visually impaired, low vision podcasts, everyday aids, contacts with, and access to, self-help groups.
7. Explanations of the physiology of the eye and brain in the pathophysiology of glaucoma.
8. Signs of vision loss and the relationship between stress and vascular dysregulation.
9. Explaining medical treatment of glaucoma and understanding the results of eye examinations.

These nine topics were each summarized in three to five individual chapters (sessions), respectively, being assigned to learning units of approx. 15 min each either in text- or video-format. The course lasted a total of nine weeks, with three to five learning units per week. At the end of each week, participants’ knowledge was tested by questionnaire to check their recall of the individual learning units. 

### Technical implementation

The web design was based on WordPress.org with its various plugins which fulfilled the following requirements: (i) it supports course function with the possibility to create video, text and audio content, (ii) member area to check the progress of participants, (iii) contact function in case problems occur during the course, (iv) availability of screen-reader function so patients with severe vision loss could participate, (v) options to change the font size and color, and (vi) a quiz function to evaluate any gain in vision related knowledge. To test the functionality of the course for people with visual impairments before the start of the study, ViWi was pilot-tested with 10 glaucoma patients at the Savir-Center in Magdeburg/Germany (www.savir-center.com). Patients were asked to fill out usability questionnaire to inspire improvements to be incorporated into the final ViWi course material. 

### Study design

The efficacy of the finalized ViWi course was evaluated in a randomized, prospective study which included 34 subjects. The course provided background knowledge to help positively influence the lifestyle and mental health with the intention to stabilize or even improve vision.

Patients were only included that met the following inclusion criteria: Age 18 + yrs, presence of subjective visual impairment, sufficient computer skills to handle the digital content, and ability to read. Patient were excluded if they had a recent history of major depressive episode, schizophrenia or other psychiatric disorders. Following informed consent, the so qualified patients were randomized as determined by lot.

**Fig. 1:**
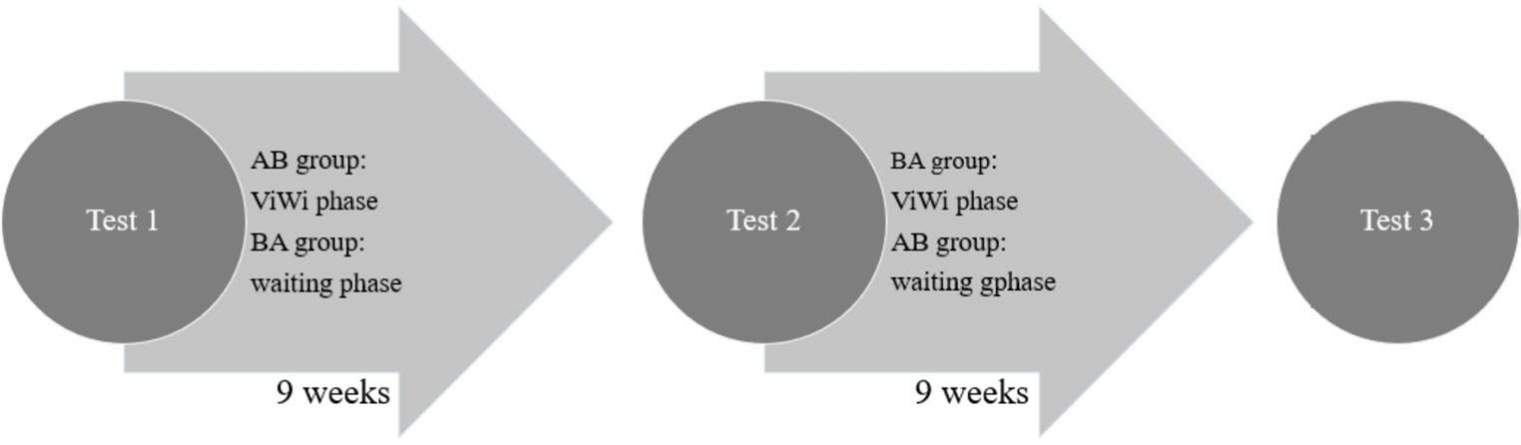
AB/BA study design showing the three testing periods

In an AB/BA study design patients were randomized to either an AB-group completing a nine-week ViWi course (A) followed by a waiting period (B) or a BA group who had to wait before taking the course (see Fig. 1 below). Of note: patients of the BA group where informed in detail prior to study entry regarding the goal of the course (which – in hindsight – might have induced a placebo-effect). Of the 46 subjects that volunteered to participate in the study, only 34 patients with a confirmed glaucoma diagnosis were randomized (AB: *N* = 13; BA: *N* = 21). Of those included, seven eventually dropped out due to lack of compliance, acute worsening of the vision or other personal problems (3 dropouts in the AB and 4 in the BA group).

The entire study was conducted remotely by using a personal electronic device (smartphone or digital tablet) in their home environment where they to complete the course with supervision – if needed - by email, post or telephone. 

### Testing periods

We used an AB/BA design (A = ViWi, B = BA) (Fig. 1) with the following time points: T1 was the assessment at baseline of the psychological status, visual impairment and background knowledge about glaucoma and personality traits using the questionnaires specified above. While patients from the AB-group carried out the 9-week course, BA patients waited for approx. 9 weeks, when at T2 they started with ViWi, at which point they were also asked to fill out the questionnaires again (except the NEO-FFI). After another period of two months (T3), the patients received the questionnaires from the second test again. All participants completed an evaluation questionnaire after taking part in the course. 

### Psychological questionnaires

Before and after completing ViWi (A) or the waiting period (B), patients were asked to fill out questionnaires to assess visual and psychological parameters.

- GAD-7 – generalized anxiety disorder.
- PHQ-9 depressive symptoms (however, excluding question 9).
- PSQ-20 – subjective stress perception measured in four domains: worries, tension, lack of joy, and demands.
- NEO-FFI Personality questionnaire of the “big five” personality traits.
- NEI-VFQ 25 assessing subjective visual impairment.
- KLQ – a “knowledge and lifestyle questionnaire” developed by us to probe current lifestyle and the level of knowledge about glaucoma-related topics.
- A usability questionnaire – developed by us for the design of the ViWi course.

During initial screening, we excluded question 9 from the PHQ 9 (“Thoughts that you would rather be dead or do something to yourself”) because patients with severe depressive episodes were not eligible to participate and refer to it as “PHQ 8” as suggested by Kroenke et al. (2009). The NEO-FFI questionnaire was used to be able to correlate personality traits with changes in the psychological questionnaires and the NEI-VFQ 25 score over the course of the study. Subjective visual function was assessed using the NEI-VFQ-25 questionnaire; no objective visual acuity or perimetric testing was performed due to the fully remote study design. The KLQ was specifically developed by us as a survey to probe patient’s knowledge about glaucoma anatomy, pathophysiology, etiology and personal lifestyle habits. Finally, the usability questionnaire probed participants´ satisfaction with the course with an option to make suggestions for improvements. 

### Intervention using the ViWi online course

During the nine-week long ViWi intervention course, patients completed three to four learning units of approx. 15 min each/week followed by a test (10 questions) at the end of each week (total of 60–90 min per week). In total, ViWi consisted of 30 learning units during a period of approx. 9 weeks. These units provided information about medical background knowledge, i.e. physiology, pathophysiology and treatment options, practical everyday tips, and psychological aspects of coping with the disease. In addition, they received an overview of existing platforms for low vision including those in apps and other media, lifestyle recommendations, nutrition tips and advice on how to cope with the disease and especially the role of stress. The total time to complete the study for each patient was approximately six months. 

### Statistical analysis

As this was a first of its kind, exploratory study, we could not estimate the required sample size to reach significance. The overall hypothesis was that participation in ViWi would improve the results of the questionnaires as a sign of improvement in psychological well-being, knowledge and lifestyle habits, all of which could improve subjective vision. The statistical analysis included a descriptive analysis of all questionnaires separately for both groups. Depending on the type of characteristic, location and dispersion parameters, absolute and relative frequencies were calculated, and inferential exploratory statistical analyses were carried out. Firstly, the questionnaire results (pre/post ViWi) were compared for both groups separately using the Wilcoxon signed-rank test. The Friedmann test for dependent samples was used to examine the three testing points during the course (testing points 1 to 3). If changes were significant, post-hoc Dunn-Bonferroni tests were used to determine which of the testing points differed. Furthermore, the results for testing point 2 were compared between the AB and BA groups using the Mann-Whitney U test.

We also assessed the scale sum values of the individual questionnaires and the categories used for interpretation. In this case, the comparison of these categorized parameters between the AB and BA groups was carried out using chi-square tests. When analyzing the knowledge questionnaire, the McNemar test or the Wilcoxon signed-rank test was used to evaluate the effect of the course on the assessment of various tools for corresponding pairwise comparisons between the testing points, depending on the appropriate scale of characteristics. Spearman correlations were calculated to investigate correlations between the (quantitative) questionnaire results and the NEO-FFI personality traits. An error probability of α = 0.05 was selected as the significance level for all statistical tests.

## Results

This section summarizes the primary outcomes of the study, focusing on changes in subjective visual function, emotional well-being, lifestyle and usability of the ViWi intervention. 

### Safety

We did not observe any adverse events or safety concerns in any of our participants. 

### Subjective visual functions

**Fig. 2:**
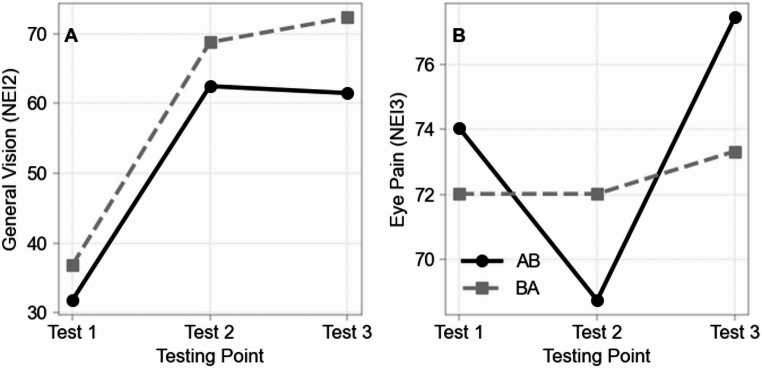
**A**, **B** Longitudinal line graph showing mean scores across the three testing points for the groups AB and BA. General vision on the left side and eye pain on the right side. Generated using Python 3.10 with pandas and matplotlib

Subjectively reported vision improved significantly in the AB-group over the course of the three test periods (χ²(2) = 16.703, *N* = 10, *p* < .001; Fig. 2a), where post-hoc testing revealed a significant improvement from the first (T1) to the second (T2) (*Z* = -1.650, *p* = .001) and third test point (T3) (*Z* = -1.350, *p* = .008), with no change from T2 to T3 (*Z* = 0.300, *p* = 1.000). The BA group (BA) also showed significant improvement in subjective vision after having completed the online course from T1 to T3 (*χ²*(2) = 23.286, *p* < .001) which was confirmed by a post-hoc comparison (*Z* = -1.667, *p* < .001). Surprisingly, the BA group also showed significant improvement from T1 to T2 even without having taken the online course (*Z* = -1.133, *p* = .006). Again, no T2-> T3 change (*Z* = -0.533, *p* = .432; Fig. 2a). Both groups were comparable in the second test point (*U* = 159.5, *Z* = 1.272, *p* = .210), i.e. significant improvements of subjective vision was surprisingly similar in both groups.

Initially, there was a statistically significant increase in eye pain during the three test phases in the experimental group (χ² (2) = 7.357, *N* = 10, *p* = .022 Fig. 2b) – more precisely between the second and third test phases, but not after adjusting the significance levels for multiple testing (*Z* = -1.000, *p* = .076). In the BA group no significant change was observed in eye pain (see Table 1). No other significant changes can be observed within the groups.

**Table 1:** Changes in subjective vision measured by the NEI VFQ 25

Subscale	AB – Group	BA- Group	Comparison of groups Testing point 2	Interpretation
General health	χ²(2) = 0.47, *p* = .846	χ²(2) = 0.05, *p* = .994	—	No change or difference between groups
General vision	**χ²(2) = 16.70**, ***p*** **< .001**	**χ²(2) = 23.29**, ***p*** **< .001**	*U* = 159.5, *Z* = 1.27, *p* = .210	Significant improvement in both groups, post hoc confirmed, no difference between groups
Eye pain	**χ²(2) = 7.36**, ***p* = .022**	χ²(2) = 1.23, *p* = .595	*U* = 156.5, *Z* = 1.20, *p* = .231	Increase in AB, not significant post hoc
Near vision	χ²(2) = 0.77, *p* = .708	χ²(2) = 0.56, *p* = .776	*U* = 173.5, *Z* = 1.79, *p* = .075 (Trend)	BA better than AB
Distance vision	χ²(2) = 0.19, *p* = .945	χ²(2) = 0.05, *p* = .994	***U* = 192.5**, ***Z* = 2.51**, ***p* = .011**	BA significantly better than AB
Social tasks	χ²(2) = 0.13, *p* = 1.000	χ²(2) = 1.60, *p* = .617	***U* = 176.5**, ***Z* = 2.08**, ***p* = .035**	BA significantly better than AB
Psych. Well-being	χ²(2) = 2.33, *p* = .340	χ²(2) = 0.49, *p* = .808	*U* = 154.0, *Z* = 1.06, *p* = .298	No differences
Social roles	χ²(2) = 2.18, *p* = .364	χ²(2) = 0.05, *p* = .993	***U* = 178.5**, ***Z* = 1.98**, ***p* = .048**	BA significantly better than AB
Dependency on others	χ²(2) = 0.86, *p* = .708	χ²(2) = 0.15, *p* = 1.000	*U* = 169.5, *Z* = 1.85, *p* = .066 (Trend)	BA trend for difference in dependency on others
Driving ability	χ²(2) =0.18, *p* = 1.000	χ²(2) = 5.74, *p* = .054	***U* = 170.5**, ***Z* = 2.52**, ***p* = .010**	BA significantly better
Color vision	χ²(2) = 2.80, *p* = .395	χ²(2) = 3.00, *p* = .667	*U* = 167.5, *Z* = 2.19, *p* = .060 (Trend)	BA trend towards better color vision
Peripheral vision	χ²(2) = 1.50, *p* = .815	χ²(2) = 5.33, *p* = .074	*U* = 156.0, *Z* = 1.18, *p* = .266	No change or difference between groups

Changes in subjective vision measured by the NEI-VFQ-25 across the three testing points; interpretation focuses on pre- to post-intervention changes (Friedman test within groups) and group comparison at T2 using Mann-Whitney U Test listed separately. Bold values indicate statistically significant results (p < 0.05). Post-hoc Dunn–Bonferroni tests were performed for significant Friedman results. Significant improvements in General Vision were observed between baseline and follow-up assessments in both groups (AB: T1–T2 *p* = .001; T1–T3 *p* = .008; BA: T1–T2 *p* = .006; T1–T3 *p* < .001). Eye Pain did not remain significant after correction

Between-group comparison at the second testing point revealed overall differences in visual functioning. The BA group performed significantly better than the AB group in several domains. However, these differences were not specific to the intervention phase, as the BA group showed consistently higher scores across at all three testing points.

**Fig. 3:**
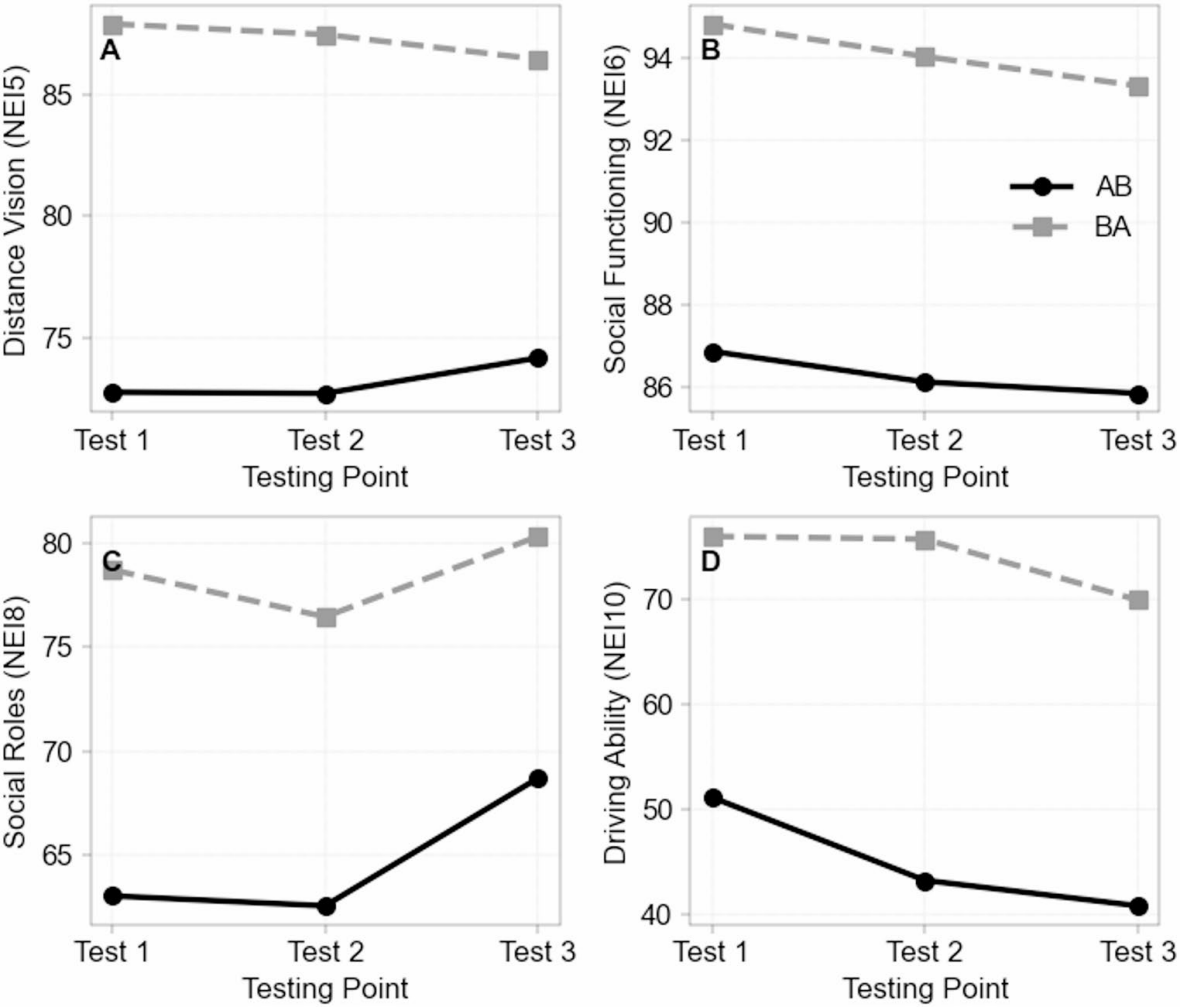
**A–D** Longitudinal line graphs showing mean scores of significant differences at testing point two. Visualized with results of all 3 testing points for AB and BA group. Generated using Python 3.10 with pandas and matplotlib

Specifically, the BA group showed a trend towards better near vision (*U* = 173.500, *Z* = 1.787, *p* = .075), significantly better peripheral vision (U = 192.500, *Z* = 2.512, *p* = .011, Fig. 3a), significantly better social functioning (*U* = 176.500, *Z* = 2.076, *p* = .035, Fig. 3b), and significantly better fulfilment of social roles (U = 178.500, *Z* = 1.977, *p* = .048, Fig. 3c), as well as a trend towards lower dependency on others (*U* = 169.500, *Z* = 1.851, *p* = .066, Fig. 3d). In addition, driving ability was significantly higher (U = 170.500, *Z* = 2.523, *p* = .010), and there was a trend towards better color vision compared with the AB group (*U* = 167.500, *Z* = 2.185, *p* = .060).

### Emotional well-being

The statistical analysis of the emotional well-being as measured by PSQ 20, PHQ 8, GAD-7 showed no statistical improvement in both groups over the course of the study (see Table 2). A trend towards a higher level of anxiety in the AB – group at testing point 1 could be detected (*U* = 173.00, *Z* = 1.77, *p* = .078).

**Table 2:** Results of the three questionnaires analyzing the emotional well-being over the course of the study:

Subscale	Experimental Group (AB)Friedman χ²(2), *p*	Control Group (BA)Friedman χ²(2), *p*	Group Comparison Testphase 2U, Z, *p*	Interpretation
PSQ – Worries	*χ*²(2) = 0.17, *p* = .951	*χ*²(2) = 0.69, *p* = .743	*U* = 162.00, *Z* = 1.36, *p* = .181	No changes; no group differences
PSQ – Tension	*χ*²(2) = 1.19, *p* = .602	*χ*²(2) = 0.69, *p* = .743	*U* = 169.00, *Z* = 1.62, *p* = .109	No significant effects
PSQ – Joy	*χ*²(2) = 0.06, *p* = .922	*χ*²(2) = 0.49, *p* = .817	*U* = 104.00, *Z* = − 0.83, *p* = .419	No changes; no group differences
PSQ – Demands	*χ*²(2) = 0.90, *p* = .686	*χ*²(2) = 2.09, *p* = .389	*U* = 154.00, *Z* = 1.33, *p* = .192	No significant effects
PSQ – Total Stress	*χ*²(2) = 1.23, *p* = .619	*χ*²(2) = 0.53, *p* = .802	*U* = 140.00, *Z* = 0.78, *p* = .445	No changes in perceived stress
PHQ-8 – Depression	*χ*²(2) = 3.16, *p* = .226	*χ*²(2) = 2.47, *p* = .305	*U* = 155.50, *Z* = 1.11, *p* = .276	No significant change; no group difference
GAD-7 – Anxiety	*χ*²(2) = 4.42, *p* = .112	*χ*²(2) = 1.04, *p* = .626	*U* = 173.00, *Z* = 1.77, *p* = .078 (trend)	**Slight trend** towards higher anxiety in AB

PSQ 20 questionnaire – analyzing the stress level in four domains. Higher scores indicate higher stress levels except for the reverse-coded subdomain “pleasure”; the PHQ 8, assessing depressive symptoms – higher scores indicating greater depressive symptoms; the GAD-7 analyzing anxiety levels - higher scores indicating greater anxiety. Results of the Friedmann test comparing the results within the groups before and after participation in ViWi on the left side and results of the Mann-Whitney U Test, comparing results of both groups in T2 on the right side

### Knowledge and lifestyle

**Fig. 4:**
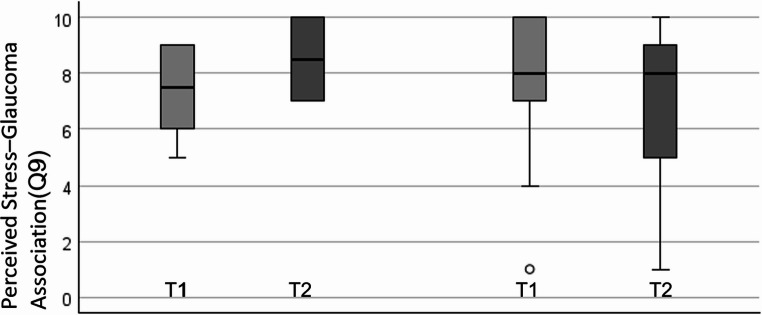
Perceived association of stress and glaucoma. This graph illustrates to what extent patients´ perception that stress and glaucoma are associated (rated on a 1–10 scale). A significant increase in the weighing of the importance of stress in terms of disease development and progression was observed in the AB-group on the left side but not in BAs (median, the upper and lower 25% IQR and the 95% CI). Visualization created using IBM SPSS Statistics

Though the analysis of the lifestyle and knowledge questionnaire showed no statistically significant improvement in lifestyle habits or knowledge about the disease after completing the online course, we noted a tendency towards a significant improvement in the subjective mental state in the BA- group (*Z* = 1.821, *p* = .074) after completion of the course, i.e. from T1 to T3 (5.07; (5.87). Also, AB-subjects perceived the association of glaucoma and stress significantly higher after completing ViWi (*Z* = 2.209513, *p* = .039) in contrast to the BA group (*Z* = 0.703, *p* = .513). However, no significant difference was observed between the groups (*U* = 60.500, *Z* = -1,447, *p* = .156) (Fig. 4).

There was a trend towards of an improvement in sleep in both groups after participation in the ViWi online course (groups AB: *Z* = 1.859, *p* = .059 and BA: *Z* = 1.872, *p* = .078) but no statistical difference between both groups in the second test (*U* = 84.500, *Z* = -1.393, *p* = .169). 

### Correlation of personality traits

NEO-FFI personality traits were comparable in both groups regarding neuroticism (*U* = 152,500, *Z* = 0.830, *p* = .417) extraversion (*U* = 114,000, *Z* = -0.591, *p* = .566), openness (*U* = 78,500, *Z* = -1.904, *p* = .057), agreeableness (*U* = 89,000, *Z* = -1.515, *p* = .134) and conscientiousness (*U* = 167,000, *Z* = 1.366, *p* = .177).

We also analyzed whether personality traits correlated with changes in the questionnaires, i.e. with ViWi efficacy. Considering the effect of personality traits on changes in the PSQ – 20 questionnaire the following was observed: Extraverted patients of the AB-group showed a trend towards negative correlation with the subscale joy over the course of test 1 to test 2 (*ρ* = -0.573, *p* = .052). In other words, extraversion was associated with a reduction in joy after participating in ViWi. In the BA group (test 1 to test 3), however, there was no correlation between personality traits and relative changes in the assessment of joy after completion of the course. In terms of the course’s influence on depressive symptoms, as measured by the PHQ 8 questionnaire, a negative correlation was found in the AB group indicating that higher compatibility was associated with a stronger reduction in depressive symptoms over the course of the study (*ρ* = -0,694, *p* = .012). In the BA group, however, no such correlation was found after completion of the course. Regarding the relative changes in the AB-group, there was a statistically significant association of higher agreeableness with higher reduction in anxiety over the course of ViWi (*ρ* = -0.730, *p* = .007), but no such correlation was found in the BA group.

Analysis of the NEI-VFQ results revealed significant associations between changes in visual functioning and personality traits. In the AB group, extraversion showed a negative correlation with changes in eye pain (*ρ* = − 0.590, *p* = .043), indicating that more extraverted participants experienced a greater reduction in eye pain over the course of the study. In the BA group, a negative correlation was found between neuroticism and the ability to perform social tasks (*ρ* = − 0.757, *p* = .002), suggesting that higher levels of neuroticism were associated with greater impairment in social functioning.

**Fig. 5:**
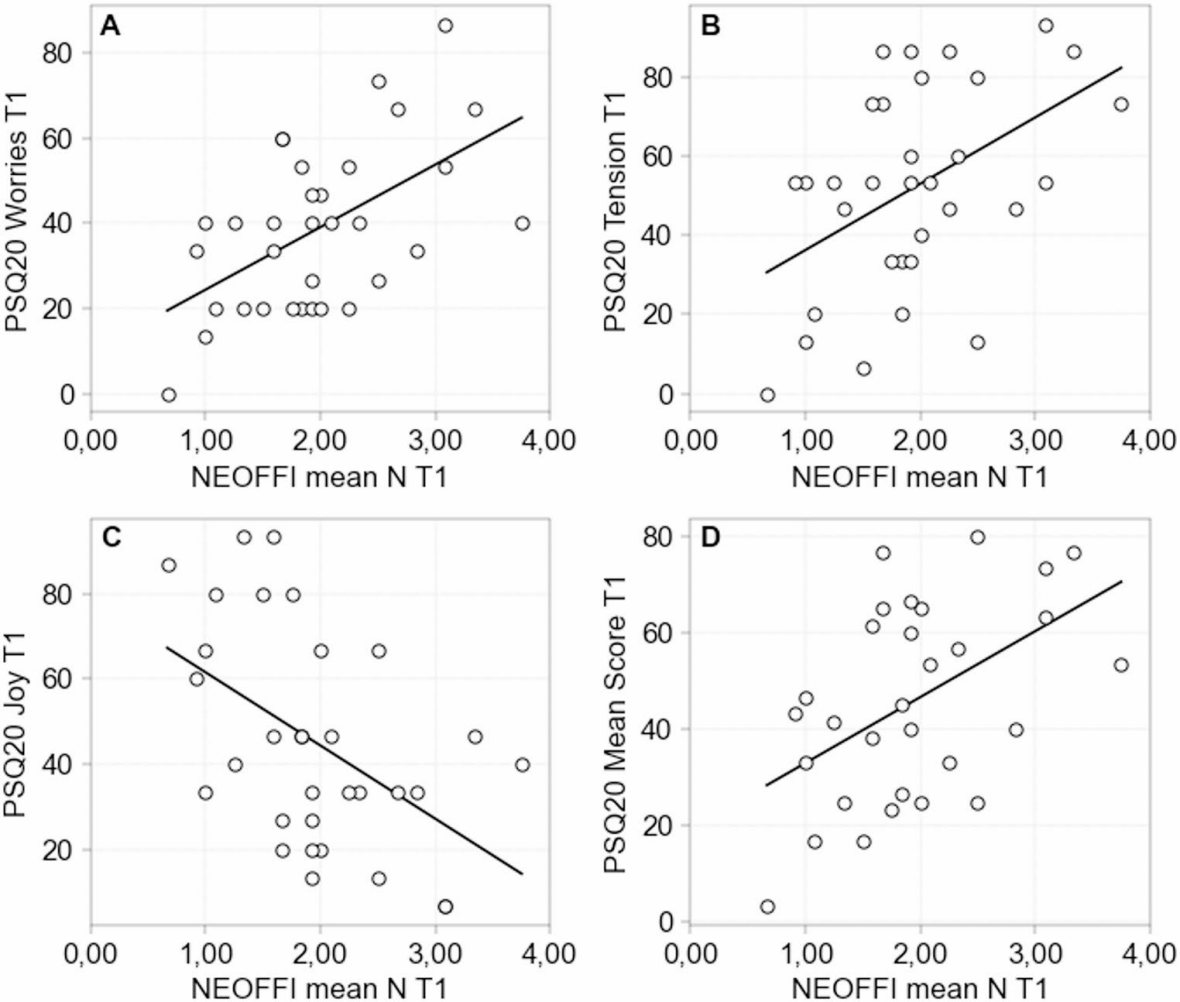
**A**–**D** Correlations between dimension N of the NEO-FFI questionnaire and the scale values for worries, tension, joy and total score of the PSQ-20 questionnaire. Visualization created using IBM SPSS Statistics

In sum, it is apparent that neuroticism correlates strongly with stress, worries, tension and reduced joy, anxiety and depressive symptoms making them extra susceptible to glaucoma (see Fig. 5).

### Usability

Both groups were comparable in their rating of ViWi usability and overall patients gave very positive feedback and rating. However, they were more critical about the technical implementation, such as the online navigating process. Overall, study participants rated as “quite good” to “very good” the website design (73%) and content (92%), and the comprehensibility “quite good” to “very good” (96%). 72% of the participants felt “quite well” to “very well” addressed by the online course and 70% said they were “quite likely” to “very likely” to recommend the course to others. However, 80% of all participants experienced technical problems during the course.

## Discussion

The aim of this exploratory study was to test if ViWi – an online-lifestyle course for glaucoma –benefits patients subjectively. To this end we analyzed the courses’ impact on various subjective levels: subjective visual function, mental well-being, how to use relaxation exercises, increase appreciation how stress management can benefit visual function [4], how greater life-style knowledge can help them cope with vision loss to learn “what else” they can do beyond standard medical care. In addition, we explored how personality traits influenced efficacy of ViWi.

In our randomized trial both groups reported that subjective vision had improved, but unexpectedly, also the BA group showed improvements already during their “waiting period”, i.e. even before the start of the ViWi course. While this could be a classic placebo effect (Fig. 2a, and discussion below), it could also be explained by BA participants searching for knowledge and new solutions online on their own (“why should I wait when advice can be found online already?”). At the end of the trial, both groups confirmed having understood how mental stress can impact eye diseases, and there was a trend towards improved sleep quality in both groups. Interestingly, agreeable subjects in the AB-group benefited most from ViWi, possibly by being able to adhere better to the instructions and remaining vigilant and active during the T2-T3 period.

Overall, despite some complaints about technical implementation of ViWi, the feedback about its value was very positive. 

### ViWi effects on subjective vision

We confirmed our key hypothesis, namely that ViWi can improve subjective vision, possibly by reducing stress, improving lifestyle habits, and fostering a sense of self-efficacy through increased knowledge about the disease and the impact of lifestyle factors on low vision [6]. Patients learned how to perform eye yoga exercises via video modules and how stress reduction (by meditation and general lifestyle adjustments) may lower intraocular pressure and increase ocular blood flow [14–20]. While patients in both groups experienced improvements in subjective vision (NEI-VFQ-25 General Vision), these changes in self-reported visual function were already observed at the second testing point (T2) (Fig. 2), including in the BA group. This finding suggests the presence of an expectancy or placebo-related effect. Although expectancy or training effects may contribute to changes in subjective visual function, they are unlikely to fully explain the observed pattern of results.

Of note, the observed changes in subjective visual function cannot be explained by repeated objective testing, as no formal visual acuity or perimetric assessments were performed. Rather, these changes may reflect functional changes in subjective visual perception, potentially mediated by stress reduction and improved vascular regulation, rather than structural restoration. One possible explanation is improved vascular regulation in regions of residual vision, leading to re-activation of functionally silent neurons, as proposed before [21].

However, both groups were quite aware of taking part in an online “lifestyle” course before they were allocated to the AB or BA group. Therefore, it is possible that patients of the BA group independently researched and scouted online for additional information and treatment options seeking advice of “what else” they could do for their glaucoma. This may have already given them a sense of self-efficacy, empowerment, relief and self-efficacy, and gaining a sense of control over their eye health. Such changes may have contributed to reduced physical and/or mental stress potentially leading to beneficial effects on IOP and blood flow [6]. It is possible that it is not a true “placebo” (expectation) effect but rather that patients became more aware of – and possibly started using – online searches to learn useful exercises and knowledge on their own, i.e. an unintended therapeutic effect while waiting for ViWi to start. To avoid such confounding effects, in future studies BA patients should not be informed of the details of an online course while waiting for the treatment phase. However, withholding this information may be unpractical and not feasible, because study participants may disagree to wait for 3 months not knowing what they wait for. 

### ViWi effects on psychological well-being

As we assumed that improved overall well-being might be a main cause for an improvement in subjective vision, we also measured stress, anxiety and depressive symptoms using validated questionnaires. Contrary to our expectation, however, there was no reduction in the perception of stress in both groups. This may have been caused by the lack of direct *in-person* contact between our team and the study participants, a major weakness of our ViWi study design. Furthermore, patient compliance could not be monitored which is why we do not know if participants performed the exercises correctly and regularly. In future studies it would therefore be useful to analyze patient compliance more closely with online user tracking or asking them to keep an online diary. Another explanation may be that the regular confrontation with issues related to their illness was an emotional burden.

This argument is in line with a study by Lange et al. [20] showing that patients who dealt intensively with their illness beyond their treatment had poorer outcomes of alleviating their symptoms. Similarly, participation in the ViWi online course did not experience reduced depressive symptoms or anxiety. These results can possibly be explained in a similar way to the lack of stress reduction: an intensive self-awareness of their own illness and the lack of further individualization of the content could have triggered negative emotions and fears in participants who did not experience an anxiety reduction. Some participants even commented that content dealing with the progression of vision loss in particular caused anxiety even in those that were only slightly impaired. For further developments of ViWi, the content should therefore be tailored in a personalised way as a function of vision loss severity and/or personality disposition. A practical solution could be the use of a chatbot surveying individual patient features before starting a ViWi online course to individually adapt it to the patient´s personal state of mind, such as personality, knowledge-level, or fear of going blind. 

### ViWi effects on lifestyle and knowledge

Another important point to be investigated in this study was the extent to which an online course can help close knowledge gaps and ensure access to evidence-based information for glaucoma patients. In everyday clinical practice, there is usually not enough time to answer all questions. Many patients therefore feel insufficiently informed [21] and try to find answers using internet research. This highlights the need to provide evidence-based online knowledge platforms like ViWi.

Despite the lack of stress reduction, ViWi increased awareness of psychological risk factors. Subjects of the AB-group, not the BA group, rated the association of stress and glaucoma as greater after completing ViWi (see Fig. 4). Furthermore, AB-patients showed a trend towards a subjective improvement in sleep quality which is positive because it is known that in glaucoma reduced sleep quality has a known negative effect on subjective well-being [22, 23] as found in the Flammer syndrome, where prolonged sleep onset time is a hallmark of glaucoma [24, 25] as also reported by Moreno-Montañés et al. [19]. This could explain why ViWi-induced sleep improvement could have a positive impact on quality of life.

### The role of personality traits

In our exploratory study we were also interested whether personality traits influenced outcome measures of our ViWi course. We observed a striking reduction of anxiety and depressive symptoms in subjects with an “agreeable” personality in the AB-group (*ρ* = -0.730, *p* = .007). Agreeable subjects, characterized by a good natured personality and willingness to cooperate, might have been particularly engaged and compliant in our online course with the effect of a more pronounced reduction in anxiety and depressive symptoms.

Another lesson learned from our study is that neuroticism as a possible risk factor because neurotic subjects suffer significantly more from stress: they worry more, are more tensed, feel less joy and suffer more frequently from anxiety and depressive symptoms. Pronounced neuroticism is partly genetic and typically correlates with negative affect and withdrawal-oriented behaviour [26]. As Sabel et al. [27] reported lower levels of neuroticism are associated with a slower progression of the eye condition. This is in line with the present study which shows that especially neurotic patients should be educated how stress and anxiety can negatively affect disease progression. Therefore, in future studies neuroticism could serve as one indicator to personalize ViWi content to patients who are particularly susceptible to stress and anxiety.

### Limitations

Because our exploratory study was the first of its kind, much was learned that is novel about this kind of behavioral intervention. But there are also numerous limitations that can inspire future study design. One limitation is that our “subjective vision improvement” was not accompanied by improvements in other subjective vision categories such as difficulties reading in poor lighting or blurred vision, both of which are included in the general vision category. Because we only included patients in our study with good residual vision in early stages of glaucoma, a “ceiling effect” may have reduced the potential of improving their visual functions. This assumption is reinforced by the fact that participants rated their general vision as very poor at the start of the study, while overall they were only slightly impaired according to the NEI VFQ 25. Yet, another limitation is that the subjective visual performance of the BA group tended to be higher than that of the AB-group. In future studies this should be avoided by appropriate baseline matching of both groups.

Yet, there is another limitation of our sampling methods: participants were recruited from the databank of the Institute of Medical Psychology at the Magdeburg University Hospital and from self-help groups, both of which represented a rather homogeneous group with presumably broad background knowledge about the disease. This could have led to a type-2 error. This may in part explain also why disease-related knowledge did not improve in both groups. Future studies should consider using a validated knowledge questionnaires to capture more sensitively the gain of knowledge and an adaptation of lifestyle and help prevent a ceiling effect.

## Conclusions

Our study demonstrates that an online lifestyle course can help glaucoma patients manage disease progression. While the function of an online course does not replace standard medical care, it is a supplemental option when patients ask: “what else can I do for my glaucoma”. The overall positive feedback from our patients illustrates how ViWi resonated well with them. Even if the improvement in subjective vision may have been in part a placebo effect, the option to participate in an online course can help increase knowledge and awareness of the important role of mental state. Specifically, the emotional relief of being in “control” and being able to do “something else” can help patients improve well-being and reduce mental stress and anxiety. It is this kind of subtle “relaxation response” that benefits the physiology of glaucoma by improving eye pressure and vascular dysregulation. We believe that this is why subjective vision also improved in both groups: the increased awareness of the risk factor “stress” and a trend towards improved sleep quality in the AB-group. While regular online sessions take time and effort, ViWi gives patients a sense that they can have some control over the situation rather than being a fearful and doomed victim that they “might” or “will” go blind. And there are no risks of adverse events.

In future studies ViWi adaptations of the course-content should be tailored more specifically to patients´ individual needs towards a personalised therapy plan. Our study could serve as a basis for planning a study with a larger and possible more heterogeneous group of patients to ascertain more sensitive and controlled testing conditions and optimizing the study design, including baseline-matching to reduce floor- and ceiling effect and adjust the design to a double-blind study to avoid “placebo” effects.

Non-medical approaches such as ViWi may represent a valuable complement to current glaucoma care by enabling patients to become more active partners in their disease management. Stress and anxiety reduction, increased relaxation. A growing sense of control may support psychological well-being and contribute to physiological mechanisms relevant to glaucoma, including the reduction of tissue and ocular tension and vascular dysregulation. In this context, psychological factors may facilitate the reactivation of silent (inactive) neurons [2, 28, 29], thereby supporting partial recovery or restoration of visual function.

Our exploratory study also unveiled associations how personality traits and psychological factors influence visual outcomes, thereby contributing to the identification and confirmation of psychosocial risk constellations relevant to glaucoma management, which is in line with the existing literature.

In sum, our ViWi approach is a practical approach of predictive, preventive and personalised medicine (PPPM). It is a preventive, person-centred digital lifestyle intervention that supports patients´ individual awareness of psychosocial and behavioural risk factors and helps them to develop strategies to address them. It is a complement to standard glaucoma care where low vision patients can learn that there is “something else” they can do to manage their glaucoma progression and achieve some vision restoration. 

## Data Availability

The data that support the findings of this study are not openly available due to reasons of sensitivity and are available from the corresponding author upon reasonable request. Data are located in controlled access data storage at the Institute of Medical Psychology, Medical Faculty, Otto-von-Guericke University of Magdeburg. All data were collected pseudonymized and analyzed in accordance with GDPR regulations.

## Declarations

### Clinical trial number

IRB 00006099 - 92/23
